# Different behavioral effects of neurotoxic dorsal hippocampal lesions placed under either isoflurane or propofol anesthesia

**DOI:** 10.1002/hipo.20390

**Published:** 2007-11-30

**Authors:** Mark G Baxter, Kathy L Murphy, Gregory Crosby, Deborah J Culley

**Affiliations:** 1 Department of Experimental Psychology, Oxford UniversityOxford, United Kingdom; 2 Department of Veterinary Services, Oxford UniversityOxford, United Kingdom; 3 Department of Anesthesia, Brigham and Women's Hospital, Harvard Medical SchoolBoston, Massachusetts

**Keywords:** hippocampus, spatial memory, learning, surgery, refinement

## Abstract

Anesthetic protocols for behavioral neuroscience experiments are evolving as new anesthetics are developed and surgical procedures are refined to improve animal welfare. We tested whether neurotoxic dorsal hippocampal lesions produced under two different anesthetic protocols would have different behavioral and/or histo-pathological effects. Rats were anesthetized with either propofol, an intravenous anesthetic, or isoflurane, a gaseous anesthetic, and multiple injections of an excitotoxin (*N*-methyl-d-aspartate) were stereotaxically placed in the dorsal hippocampus bilaterally. Intraoperative physiological parameters were similar in the two surgical groups, as were the volumes of the lesions, although the profile of postoperative impairment in a spatial learning task differed between the lesion groups depending on the anesthetic regimen used. These results show that the choice of anesthetic protocol is a critical variable in designing behavioral neuroscience experiments using neurosurgical procedures. This factor should be considered carefully in experimental design and in cross-study comparisons of lesion effects on behavior. © 2007 Wiley-Liss, Inc.

General anesthetics produce, to varying degrees, a loss of consciousness, muscle relaxation, and analgesia through a variety of different pharmacological mechanisms, some better understood than others. Anesthesia has a variety of effects, both temporary and enduring, on the function of the central nervous system. For example, 2 h of isoflurane-nitrous oxide anesthesia impairs acquisition of a hippocampal-dependent spatial memory task 48 h after cessation of anesthesia in young rats and at least 2 weeks after cessation of anesthesia in aged rats (Culley et al., 2004a,b; Crosby et al., 2005). These and other considerations imply that general anesthesia is not as benign as may be usually assumed, and that the induction of a pharmacologic coma as part of an experimental protocol—for example, to place a surgical lesion in experimental animals—is a critical variable in the procedure.

In the present study, we considered whether the anesthetic protocol could affect the neurotoxic action of a glutamate agonist in vivo. The impetus for this study was the observation that a neurotoxic lesion of the nucleus accumbens was ineffective in a single marmoset anesthetized with propofol instead of alphadalone/alphaxalone (P. Taylor, personal communication, February 2006). Propofol anesthesia may have some advantages relative to volatile anesthetics (such as isoflurane and sevoflurane) in some neurosurgical procedures because propofol does not stimulate cerebral blood flow in the way that volatile anesthetics do (Hans and Bonhomme, 2006). Thus, before pursuing the use of propofol as an anesthetic for neurosurgery in nonhuman primates, we determined whether neurotoxic lesions of a brain structure in rats (the hippocampus) differed in their behavioral and/or neuroanatomical effects in rats anesthetized either with isoflurane or propofol. Rats received neurotoxic dorsal hippocampal lesions, produced by multiple injections of the excitotoxin *N*-methyl-d-aspartate (NMDA), while under either isoflurane or propofol anesthesia. We then trained rats postoperatively, after a 14-day recovery period, in a 3/6 reference/working memory task on the radial arm maze (e.g., Schmitt et al., 2003) in which the same three of six arms were always baited on each session of testing, but the rewards were not replaced within each trial. We chose this protocol because we expected it to be straightforward enough to be completed even by rats with severe spatial learning impairments induced by dorsal hippocampal damage (Bannerman et al., 1999) and because it would produce less of a demand on spatial memory than other protocols which are sensitive enough to detect impairments in spatial memory that are induced by some anesthesia protocols, even without surgery Culley et al., 2004b).

Temperature was monitored in each rat by a rectal probe, and blood pressure was monitored via a rat tail cuff noninvasive blood pressure instrument (Powerlab, AV Instruments) in most rats. In some rats the placement of the tail vein cannula for propofol delivery interfered with blood pressure measurement and temperature. Anesthesia delivery was modified intraoperatively to maintain normotension in conjunction with other indicators of depth of anesthesia. A venous blood gas was determined in most rats ∼40 min after beginning of neurotoxin or sham injections. With the exception of body temperature, which was slightly lower in rats anesthetized with isoflurane, these parameters were not significantly different between the two groups of rats (Table 1). These parameters also indicate that we were successful at maintaining normotension and normothermia intraoperatively, and there was no evidence of hypoxia, hypercapnia, alkalosis, or acidosis. Thus, any intraoperative physiological differences attributable to different anesthetic protocols cannot account for differences in postoperative behavior, at least based on the parameters that we have measured.

**Table 1:** Intraoperative Physiological Parameters in Rats Anesthetized with Isoflurane or Propofol

	Isoflurane	Propofol	Comparison
Mean arterial blood pressure (mm Hg)	89.72 ± 2.36 (14)	93.84 ± 2.60 (8)	*t*(20) = 1.12, ns
Temperature (°C)	36.95 ± 0.09 (14)	37.37 ± 0.13 (9)	*t*(21) = 2.76, *P* = 0.012
pH	7.37 ± 0.02 (7)	7.33 ± 0.03 (12)	*t*(17) = 1.19, ns
pCO_2_ (mm Hg)	48.21 ± 3.67 (7)	56.08 ± 4.95 (13)	*t*(18) = 1.07, ns
pO_2_ (mm Hg)	65.04 ± 10.64 (7)	74.79 ± 6.99 (12)	*t*(17) = 0.80, ns

Shown as mean ± SEM (*N* for each determination, includes both lesions and controls).

The NMDA injections, as intended, ablated the dorsal hippocampus bilaterally in each of the lesion cases. We estimated the volume of the hippocampal lesions by plotting them onto standard rat brain sections (Paxinos and Watson, 2005) and measuring the volume of damage to dorsal and ventral hippocampus. Hippocampal damage was comparable in the two lesion groups, with both groups sustaining extensive ablation (∼90%) of dorsal hippocampus and minimal damage (<10%) to ventral hippocampus (Table 2). There was a trend for the propofol-lesion group to have slightly more unintended damage to ventral hippocampus, but this did not quite reach statistical significance and was a very small difference (less than 3%). Photomicrographs of representative lesions for each group are illustrated in Figure 1.

**Figure 1:**
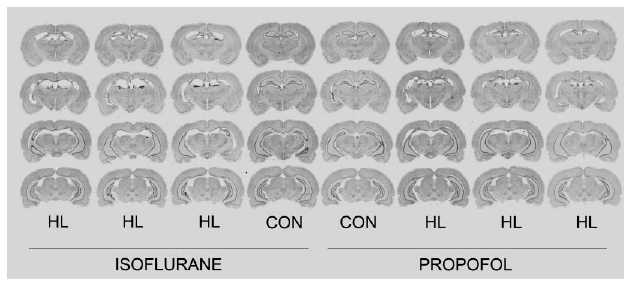
Coronal cresyl violet-stained brain sections (moving from rostral to caudal from the top to the bottom of the figure) through the hippocampus for three representative rats with dorsal hippocampal lesions (HL) from each group (isoflurane-lesion and propofol-lesion) and one control rat (CON) from each group. Each column represents a single case.

**Table 2:** Percent Loss of Dorsal, Ventral, and Total Hippocampal Volume in Rats Anesthetized with Isoflurane or Propofol

	Isoflurane	Propofol	Comparison
Dorsal	90.11 ± 1.94	91.28 ± 0.67	*t*(12) = 0.57, ns
Ventral	7.87 ± 1.01	10.48 ± 0.71	*t*(12) = 2.12, *P* = 0.056
Total	48.64 ± 1.25	50.55 ± 0.62	*t*(12) = 1.36, ns

Shown as mean ± SEM (seven per group).

We analyzed a number of behavioral parameters related to performance in the radial maze. With regard to overall behavioral performance, lesions increased the amount of time required to complete the maze (time required to obtain all three rewards, to a maximum of 10 min) overall, *F*(1, 22) = 31.78, *P* < 0.0005. Time to complete the maze decreased across 3-day training blocks across all rats, *F*(6, 132) = 55.65, *P* < 0.0005, but this effect did not interact with lesion, *F*(6, 132) = 1.47, *P* = 0.20, nor was there a main effect of anesthesia condition or an interaction of anesthesia with any of these effects, *F*s < 1. However, when we analyzed the total number of arm entries made, there were main effects of lesion, block, a block = lesion interaction, and a 3-way interaction of block, lesion, and anesthetic, *F*(6, 132) = 6.65, *P* = 0.047. These data are plotted in Figure 2. Post hoc analysis with focused ANOVAs revealed no effects of anesthetic or block in sham rats, *P*s < 0.17, but significant effects of block, *F*(6, 72) = 3.60, *P* = 0.004, and a block × anesthesia interaction, *F*(6, 72) = 2.23, *P* = 0.05, in the lesioned rats. Evaluation of the lesion × anesthetic interaction for each block separately (Howell, 2007) revealed significant interactions for blocks 3 and 7, *F*s(1, 22) = 4.33 and 8.17, *P*s = 0.047 and 0.009, respectively. This interaction seems to stem from two effects: the propofol-lesion group makes more entries relative to propofol-sham rats at an intermediate point in training, and the isoflurane-lesion group makes more entries relative to isoflurane-lesion rats at a later point in training. Although the effect does not reach statistical significance, it is also clear that isoflurane-lesion rats are making fewer arm entries early in testing. Thus, the behavior of the lesioned rats on the maze depends on the anesthetic under which they were operated.

**Figure 2:**
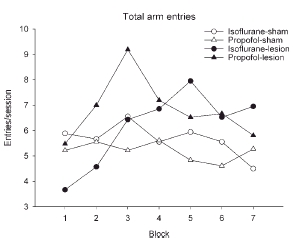
Number of arm entries for each 3-day block of testing for rats in each group. There is a significant three-way interaction of anesthetic, lesion, and block. Propofol-lesion rats make more entries at intermediate stages of testing because they are committing more errors; the same is true for isoflurane-lesion rats at the end of testing. There are no differences between the control groups at any point. isoflurane-lesion rats also make fewer arm entries early in testing.

Few arm entries early in training in the isoflurane group could reflect lower motivation in this group. Time taken to retrieve and eat a Fruit Loop given in the home cage was recorded after completion of day 2 of behavioral testing. These measures did not show significant effects of lesion, *F*s(1, 22) < 1.31, *P*s > 0.26, anesthetic condition, *F*s(1,22) < 0.79, *P*s > 0.38, or their interaction, *F*s(1, 22) < 1, *P*s > 0.40. At least based on this measure, it does not seem that gross differences in motivation to consume the rewards can account for differences in exploratory behavior.

Because of the differences in the number of arm entries at different phases of training, we analyzed a composite percent correct score as the primary measure of maze performance, dividing the number of rewards obtained (0–3) by the total number of arm entries (including correct choices and all errors). This adjusts, to some degree, for effects early in training where isoflurane-lesion rats are making few errors because they are also making few choices. These data are shown in Figure 3. Both sham groups gradually improve their performance across training. Both lesion groups are impaired, as would be expected, but the impairment is apparent earlier in training in the propofol-lesion group, and appears more severe overall. Analysis of this measure reveals a main effect of lesion, *F*(1, 22) = 36.65, *P* < 0.0005, a main effect of block, *F*(6, 132) = 2.60, *P* = 0.02, and, critically, a 3-way interaction of block, lesion, and anesthetic, *F*(6, 132) = 2.42, *P* = 0.03. We also noted three-way interactions of block, lesion, and anesthetic on errors of commission, *F*(6, 132) = 2.25, *P* = 0.042, and on reference memory errors, *F*(6, 132) = 2.90, *P* = 0.011. These effects resemble the result with arm entries: the lesion effect in rats anesthetized with propofol (relative to the lesion effect in rats anesthetized with isoflurane) is largest at an intermediate point in training, whereas the lesion effect in rats anesthetized with isoflurane (relative to the lesion effect in rats anesthetized with propofol) is largest at the end of training.

**Figure 3:**
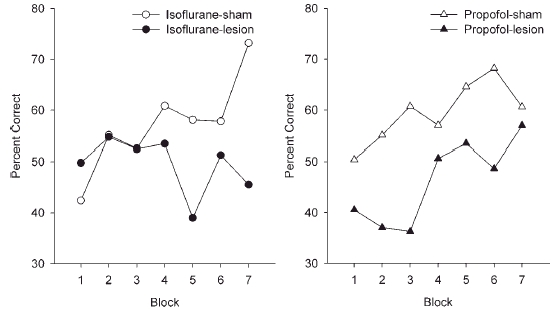
Percent correct measure of performance (number of rewards retrieved divided by number of arm entries) for each group. This measure was chosen because of the effects of lesion and anesthetic condition on number of arm entries, in an attempt to adjust for effects of the lesions on maze exploration. There is a significant three-way interaction of anesthetic, lesion, and block. The impairment in the propofol-lesion group appears to emerge earlier in testing, and is more severe at this point, relative to the impairment in the isoflurane-lesion group, which emerges toward the end of testing.

Thus, differences in the postoperative behavior of rats with dorsal hippocampal lesions were noted when they were tested in the radial arm maze, depending on whether they had been anesthetized with isoflurane or propofol during surgery. This appeared to be related to differences in the time the maximal lesion effect on performance appeared during the course of postoperative testing, as well as an initial suppression of exploration of the maze in the isoflurane-lesion group relative to the propofol-lesion group. No differences were seen in the behavior of sham-operated rats depending on anesthesia condition. These results show that the pattern of postoperative impairment in spatial learning following dorsal hippocampal lesions—a very reliable finding in the experimental literature—can be affected by differences in the anesthetic regimen used during the surgical procedures to produce the lesions. This is true even though we did not notice obvious differences in the lesions between the two anesthetic groups, and the volume of hippocampal damage was the same in both groups.

Reasonable measures were taken to ensure that other differences between the groups could not explain the results observed. Intraoperative physiological parameters were not significantly different between the two groups, with the exception of a small difference in body temperature, and the hippocampal lesions were similar between the groups. All rats were subjected to the same food restriction and training procedures contemporaneously. Similarly, the lesions were performed by the same surgeons, and administration of anesthesia was monitored by the same individuals for all groups. Thus, the differences observed do not represent a cohort effect by testing, for example, rats operated under isoflurane at one time and rats operated under propofol at another.

The different lesion effects in different groups may relate to interactions of the NMDA neurotoxin with the anesthetics, the anesthetics with postoperative diazepam treatment to prevent seizures (diazepam potentiates GABA action at GABA_A_ receptors; Rudolph et al., 2004), or a complex interaction of all three. Notably, the two anesthetics we chose in the present study have some different mechanisms of action; isoflurane, like other inhalation anesthetics, appears to have a number of effects including hyperpolarization of neurons via increasing potassium conductances as well as modulation of multiple ligand-gated ion channels, including the GABA_A_ receptor (Evers and Koblin, 2004), whereas propofol is a relatively selective modulator of the GABA_A_ receptor (Harrison and Sear, 2004). It may be possible to dissociate these effects by, for example, conducting a similar experiment with radiofrequency hippocampal lesions, which damage hippocampal tissue directly and do not rely on excitotoxicity, and thus may also reduce or eliminate the need for postoperative treatment with diazepam.

These findings may have specific implications for testing designs in which behavior is sampled at a limited number of time points postoperatively—for example, one. If the difference between lesioned and control rats differs at different postoperative times depending on the anesthetic chosen, this could introduce a significant source of variability between studies in different laboratories, or between experiments in the same laboratory if anesthetic procedures are modified. This is not often considered as a factor that could contribute to inter-study variability. We stress that this should not be used as a reason to avoid refinement and improvement of anesthetic protocols, when this is possible. It does mean that changes in anesthetic regimen need to be considered as a factor in experimental outcomes. Thus, we would advocate formalized testing of effects of changes of anesthetic regimen on the behavioral effects of brain lesions, when changes in anesthetic protocols are contemplated.

Some anesthetics have been associated with cognitive impairment in the absence of any surgical manipulation, for example isoflurane administered with 70% nitrous oxide (Culley et al., 2004a,b). Post-anesthetic cognitive impairment is exacerbated in aged rats, who already have compromised neural systems that are involved in cognitive functions such as spatial learning. It is possible that the surgical destruction of the hippocampus with NMDA injections in the present study also reveals some postanesthesia effects on cognition that are not evident in young rats that received sham surgeries (or no surgery). Thus, like chronological age, hippocampal damage may produce impaired central nervous system function that renders the organism more vulnerable to deleterious cognitive and neurobiological effects of general anesthesia. Although it is not possible to separate the effects of the hippocampal lesion from any effects of anesthesia in the current experimental design, future experiments could examine the effects of additional episodes of administration of different anesthetics on rats that had already received hippocampal lesions placed under a single anesthetic regime.

## DETAILED METHODS

All experimental procedures were approved by the Harvard Medical Area Standing Committee on Animals and were performed in laboratories at Brigham and Women's Hospital, Department of Anesthesia, Boston, MA.

### Subjects

Twenty-eight male Fischer-344 rats, 4.5 months old (292–366 g at the time of surgery) were singly housed in polycarbonate cages in a colony room with automatically regulated lighting (12/12 h light/dark cycle, lights on at 0700). Food and water were available ad libitum until 10 days before behavioral testing, at which point daily food intake was restricted to bring body weights to 85% of ad libitum levels.

### Surgery and Anesthesia

Fourteen days before beginning behavioral testing, rats were randomly assigned to one of the four groups: isoflurane-lesion (*N* = 8), isoflurane-sham (*N* = 6), propofol-lesion (*N* = 8), or propofol-sham (*N* = 6). Each rat was weighed and anesthesia was induced with 3.5% isoflurane in 100% oxygen in an induction chamber. Rats in the isoflurane groups remained on isoflurane once anesthesia was induced, administered through a mask or nosecone mounted on the stereotaxic frame (1.75%–4%, to effect). A tail vein cannula was placed in each rat in the propofol groups within 3–4 min of induction with isoflurane, propofol infusion began (0.6–0.9 mg/kg/min), and isoflurane was reduced to 0.5% and subsequently discontinued. This rate of infusion for propofol corresponds well to that determined from propofol effects on EEG parameters (ED_50_ of 0.73 mg/kg/min; Tzabazis et al., 2004). When a surgical plane of anesthesia was induced via propofol infusion alone the rat was moved to the stereotaxic frame, and continued to receive 100% oxygen via a nosecone mounted on the stereotaxic frame. In both groups of animals, the head was shaved and carprofen (5 mg/kg, s.c.) administered immediately prior to placement in the stereotaxic frame. Surgical anesthesia was monitored by a lack of responsiveness to toe or foot pinch, respiratory rate, and a lack of responsiveness to surgical stimulus when present.

The head was cleansed with Betadine and alcohol and the eyes were covered with petroleum jelly to prevent dehydration of the corneas. The skin over the skull was cut, the skull was leveled between bregma and lambda, and small burr holes were drilled over the sites into which the 30-gauge needle of a 10-μl Hamilton syringe would be introduced (Five in each hemisphere targeting the dorsal hippocampus, following Glenn and Mumby, 1998: AP ± 3.1, ML ± 1.0, DV −3.6; AP −3.1, ML ± 2.0, DV −3.6; AP −4.1, ML ±2.0, DV −4.0; AL −4.1, ML ±3.5, DV −4.0; AP −5.0, ML ±3.0, DV −4.1, mm relative to Bregma). For rats in the lesion groups, the needle was lowered to the appropriate DV coordinate and 0.4 μl NMDA (0.09 M) was infused at 0.15 μl/min. The needle remained in place for 140 s after each infusion (for a total dwell time of 5 min/site). For rats in the sham groups, the needle was lowered 2 mm and left in place for 5 min. At the end of surgery, the wound was sutured with absorbable sutures (Vicryl), and saline (0.9%, 2 ml s.c.) and diazepam (1 mg/kg, i.m.) were given to provide hydration and prevent seizures, respectively. Isoflurane anesthesia continued for 10–20 min and was withdrawn gradually; propofol anesthesia was discontinued when sutures were placed. Rats were then placed in a 100% oxygen atmosphere until they showed signs of wakefulness, at which point they were returned to their home cages. Carprofen was given again 12, 24, 36, and 48 h postoperatively (2.5 mg/kg, s.c.) for analgesia.

To ensure comparability of results, the surgical procedures under the two anesthetic protocols were performed at the same time by the same two surgeons, the two individual stereotaxic frames used were counterbalanced across anesthetic protocols, and all postoperative behavioral testing was conducted by individuals blinded to treatment condition.

### Radial Arm Maze Testing

Testing procedures were adapted from Culley et al. (2004b) and were conducted with the same 12-arm radial maze apparatus. In an attempt to simplify the task so that performance of rats with hippocampal lesions was not at floor, possibly obscuring differences between the lesion groups, we removed every other arm of the maze, converting it to a 6-arm maze, and confronted the rats with a 3/6 working/reference memory task. Three of the 6 arms of the maze (arms 3, 5, and 6; the same for all the rats) were designated “reference memory arms” and were never baited. The remaining three arms contained a food reward (a Fruit Loop). On days 12–14 postoperatively, rats were placed in the maze and allowed to explore it freely for 10 min and collect rewards scattered about the maze.

Beginning on postoperative day 15, working/reference memory testing began and continued for 21 days. The rat was placed in the center of the maze and was allowed to explore it until all three food rewards were collected. Entries into each arm, defined as all four paws of the rat proceeding two-thirds of the way down an arm, were recorded. Errors were scored in several categories: reference memory errors, first entries on each day into a reference memory arm; errors of commission, repeat entries into arms that had been visited before on that session (i.e., working memory errors), and errors of omission, which represented failures to obtain a reward within the time limit of testing (10 min for each daily session). Time to complete the maze was also recorded. Because rats varied in the extent to which they explored the maze, we also analyzed a composite percent correct measure which was the number of correct choices (rewards retrieved) divided by the total number of choices made (the sum of the number of rewards retrieved/reference memory errors, and errors of commission). Data were analyzed by parametric analysis of variance (ANOVA) with three factors, one within-subject (testing block, composed of average performance across 3 consecutive days of testing, forming seven blocks), and two between-subject, anesthetic condition (propofol or isoflurane), and lesion (sham or hippocampal).

One isoflurane-lesion rat would not run the maze reliably and was eliminated from the study, and one propofol-lesion rat was euthanized before testing began because of poor wound healing from the surgery, leaving seven rats in each lesion group.

### Histology

Rats were given a lethal dose of sodium pentobarbital (100 mg/kg) and transcardially perfused with 0.9% saline followed by 10% formalin. Brains were stored in formalin and cryoprotected in formalin–sucrose before being sectioned coronally at 50 μm on a freezing-sliding microtome. Sections were mounted on gelatin-coated slides and stained with cresyl violet.

## References

[b1] Bannerman DM, Yee BK, Good MA, Heupel MJ, Iversen SD, Rawlins JN (1999). Double dissociation of function within the hippocampus: A comparison of dorsal, ventral, and complete hippocampal cytotoxic lesions. Behav Neurosci.

[b2] Crosby C, Culley DJ, Baxter MG, Yukhananov R, Crosby G (2005). Spatial memory performance 2 weeks after general anesthesia in adult rats. Anesth Analg.

[b3] Culley DJ, Baxter MG, Crosby CA, Yukhananov R, Crosby G (2004a). Impaired acquisition of spatial memory 2 weeks after isoflurane and isoflurane-nitrous oxide anesthesia in aged rats. Anesth Analg.

[b4] Culley DJ, Baxter MG, Yukhananov R, Crosby G (2004b). Long-term impairment of acquisition of a spatial memory task following isoflurane-nitrous oxide anesthesia in rats. Anesthesiology.

[b5] Evers AS, Koblin DD, Evers AS, Maze M (2004). Anesthetic Pharmacology: Physiologic Principles and Clinical Practice.

[b6] Glenn MJ, Mumby DG (1998). Place memory is intact in rats with perirhinal cortex lesions. Behav Neurosci.

[b7] Hans P, Bonhomme V (2006). Why we still use intravenous drugs as the basic regimen for neurosurgical anaesthesia. Curr Opin Anaesthesiol.

[b8] Harrison NL, Sear JW, Evers AS, Maze M (2004). Anesthetic Pharmacology: Physiologic Principles and Clinical Practice.

[b9] Howell DC (2007). Statistical Methods for Psychology.

[b10] Paxinos G, Watson C (2005). The Rat Brain in Stereotaxic Coordinates.

[b11] Rudolph U, Crestani F, Möhler H, Barr J, DeLorey TM, Lameh J, Davies MF, Evers AS, Maze M (2004). Anesthetic Pharmacology: Physiologic Principles and Clinical Practice.

[b12] Schmitt WB, Deacon RM, Seeburg PH, Rawlins JN, Bannerman DM (2003). A within-subjects, within-task demonstration of intact spatial reference memory and impaired spatial working memory in glutamate receptor-A-deficient mice. J Neurosci.

[b13] Tzabazis A, Ihmsen H, Schywalsky M, Schwilden H (2004). EEG-controlled closed-loop dosing of propofol in rats. Br J Anaesth.

